# Effect of Single-Walled Carbon Nanotubes on the Cross-Linking Process in Natural Rubber Vulcanization

**DOI:** 10.3390/polym15010126

**Published:** 2022-12-28

**Authors:** Yoliria Vázquez-Martínez, Claudia A. Ramírez-Herrera, Margarita Mondragón, Alex Elías-Zúñiga, Luis E. Elizalde

**Affiliations:** 1 Departamento de Química Macromolecular y Nanomateriales, Centro de Investigación en Química Aplicada (CIQA), Blvd. Enrique Reyna Hermosillo 140, Saltillo 25294, Coah., Mexico; 2 Institute of Advanced Materials for Sustainable Manufacturing (IAMSM), Department of Mechanical Engineering and Advanced Materials, Tecnologico de Monterrey, Av. Eugenio Garza Sada 2501 Sur, Monterrey 64849, N. L., Mexico; 3 Instituto Politécnico Nacional, Centro Interdisciplinario de Investigación para el Desarrollo Integral Regional (CIIDIR), Santa Cruz Xoxocotlán 71230, Oax., Mexico

**Keywords:** natural rubber, vulcanization, single-walled carbon nanotubes, organic peroxides, nanocomposites, mechanical properties

## Abstract

In this study, the effect of single-walled carbon nanotubes (SWCNTs) on the cross-linking of natural rubber (NR) using organic peroxides was investigated. NR-SWCNTs nanocomposites were prepared in an open two-roller mill followed by vulcanization with the compression molding process. Three different organic peroxides, 1,1-bis(tert-butylperoxy)-3,3,5-trimethylcyclohexane (T29), dicumyl peroxide (DCP), and 2,5-bis(tert-butylperoxy)-2,5-dimethyl-3-hexyne (T145), were used as vulcanizing agents. SWCNTs promote a remarkable reduction in the vulcanization time and increase the degree of cross-linking of vulcanized rubber when compared with neat or natural rubber–carbon-black composites; the same tendency was obtained in the NR-SWCNTs vulcanized with sulfur. Additionally, the mechanical performance of the NR-SWCNTs composites was significantly improved up to 75, 83, 27, and 10% for tensile strength, moduli, tear strength, and hardness. Raman spectroscopy studies evidence the occurrence of reaction between nanotube walls and free radicals generated from using organic peroxides during the vulcanization process. These results demonstrate that the incorporation of SWCNTs in combination with the use of organic peroxides for the NR vulcanization represents a potential alternative for the improvement of the physicochemical properties of NR composites.

## 1. Introduction

Natural rubber (NR) is an elastomer capable of recovering its shape from large deformations quickly and forcibly [1]; additionally, it possesses many other unique properties such as: (i) low elastic modulus and high percent of elongation before failure, (ii) very low thermal conductivity, and (iii) exhibiting significant hysteresis under cyclic loading, which contributes to its energy absorption capability. These unique properties have led many industries to adopt NR for a wide range of products in an endless number of applications, from the simplest systems to the most complex, from home products to the aerospace industry. 

To improve its mechanical properties, NR needs to be formulated and vulcanized; this process involves the transformation of a plastic rubber compound into a highly elastic end-product with the formation of chemical cross-links between rubber chain segments that build a 3D network within the rubber matrix. Vulcanization is performed by the addition of cross-linking agents such as sulfur, fillers, lubricants, and stabilizers. Another possibility is the use of organic peroxides as cross-linking promoters [2,3]. Worldwide, sulfur is the most frequently used vulcanizing agent for NR due to its ability to form covalent sulfur bonds between the elastomeric chains, which give the sulfur vulcanizate excellent elastic and dynamic properties, and high tensile and tear strength. However, NR products vulcanized with sulfur exhibit some drawbacks such as their low heat-aging resistance and poor thermo-oxidative stability [4,5]; additionally, once reaching their useful life, it is well known that these materials cannot be burned due to their sulfur content, so if not properly handled, they can create serious environmental problems [6].

In that sense, to avoid the use of sulfur as a cross-linking agent, organic peroxides represent a relatively simpler alternative to vulcanize both unsaturated and saturated rubbers. Thermal decomposition of the organic peroxides produces free radicals during the vulcanization, which react with the double bonds present in the NR, resulting in cross-linked polymer chains. C-C cross-links exhibit higher thermal stability than sulfur bonds; therefore, NR compounds vulcanized with organic peroxides possess good heat-aging resistance, high thermal stability, good electrical properties, and no staining of the end-products. Despite the benefits, NR vulcanized with organic peroxides usually exhibits lower elastic and dynamic properties such as tensile and tear strengths than NR vulcanized with sulfur [3,5,7].

The addition of fillers to rubber compounding formulation has been used to improve the physicochemical properties of NR compounds vulcanized with organic peroxides. In most cases, the fillers act as a reinforcing material, interacting with the elastomer matrix. The strong influence of relatively small amounts of filler particles as carbon black (CB) on the mechanical properties of elastomers has been well known for decades and has significantly contributed to the increasing use of elastomeric materials in many commercial applications [8,9,10]; silica particles also have been extensively used for this purpose [11,12]. However, interactions among elastomeric systems can be further improved by increasing the surface area to volume ratio; so, the use of smaller particles of fillers with high aspect ratios is a common practice to enhance the composite moduli values, failure properties (tensile and tear strength), and abrasion resistance. Based on this concept, several types of nanoparticles have been used as fillers, such as nanoclays [13,14], nanofibers [15,16], or carbon nanotubes (CNTs) [17,18,19,20,21,22,23,24,25,26]. Particularly, the incorporation of CNTs into rubbers has been identified as one of the more suitable alternatives to substitute the traditional use of CB particles [27], since they provide enhanced stiffness (i.e., enhance the initial stiffness and retain the stiffness enhancement for large overall strain deformation behavior) while also retaining the important attributes of [28,29].

NR filled with single-walled carbon nanotubes (SWCNTs) and cross-linked with sulfur has been previously reported in the literature. López-Manchado et al. [17] reported the incorporation of 10 phr of SWCNTs without modification or previous treatment in NR, highlighting that SWCNTs reduced the energy required for cross-linking. Zhao et al. [18] analyzed the use of SWCNTs as sensors in the vulcanization of NR by measuring the Raman spectra. Anand et al. [19] found that the addition of very low loads of SWNTs (~2 phr) to an NR matrix can provide effective reinforcement and improved electrical properties in NR nanocomposites obtained by a latex-stage mixing method. The enhanced reinforcement observed in NR-SWCNTs nanocomposites was attributed to the effective filler–rubber interactions achieved from an adequate SWCNTs dispersion in the rubber matrix, resulting in additional cross-links in the elastomeric network. Nevertheless, to our knowledge, the effect of SWCNTs on the vulcanization process of NR systems from using organic peroxides has yet to be reported in the literature. Therefore, we report the study of the vulcanization process of NR using organic peroxides and their interaction with very low SWCNTs content (<1 phr) as a reinforcing agent from the analysis of the mechanical properties and Raman spectroscopy. These results are compared with NR composites obtained using sulfur as a cross-linker and CB as filler.

## 2. Materials and Methods

### 2.1. Materials

**Figure 1:**
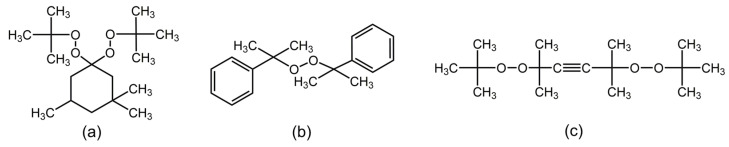
Chemical structure of organic peroxides used as cross-linking agents: (**a**) T29, (**b**) DCP, and (**c**) T145.

NR was purchased from Elastómeros y Polímeros Sintéticos S.A. de C.V. (Tlalnepantla de Baz, Mexico). SWCNTs with an average diameter of 10 nm and lengths about 10–30 μm were purchased from Bucky USA (Houston, TX, USA); they were used without any previous purification. CB DARCO G60 particles were obtained from ICI America Inc. (Wilmington, DE, USA). Stearic acid (95%), bis(benzothiazyl) disulfide (MBTS, 99%), phenyl beta naphthyl amine (PBN, 97%), zinc oxide (97%), xylene (99%), and sulfur were purchased from Sigma Aldrich (St. Louis, MO, USA) and used as received. Three organic peroxides obtained from Akzo Nobel Chemicals (Dobbs Ferry, NY, USA) were used in this study: 1,1-bis(tert-butylperoxy)-3,3,5-trimethylcyclohexane (T29), dicumyl peroxide (91%) (DCP), and 2,5-bis(tert-butylperoxy)-2,5-dimethyl-3-hexyne (T145). The chemical structure and some physical properties of the organic peroxides used in this work are shown in Figure 1 and Table 1, respectively.

### 2.2. Dispersion of SWCNTs in NR

To improve the dispersion of SWCNTs in NR, a masterbatch of SWCNTs was prepared. Five grams of NR were dissolved in 40 mL of xylene; separately, 0.7 g of SWCNTs were dispersed by ultrasonication in 50 mL of xylene using an ultrasonic bath, Model 3510 (Branson Ultrasonic Corporation, Brookfield, CT, USA) for 20 min at 6 W. Both dispersions were mixed and sonicated for an additional 30 min. Then, the solvent was removed at 110 °C with continuous agitation. The SWCNTs masterbatch was used as a source of SWCNTs for the nanocomposites prepared in this work.

### 2.3. Preparation of NR Composites with Sulfur

NR composites using sulfur as cross-linker and SWCNTs or CB as fillers were prepared in an open two-roll mill Polymix 80T (Schwabenthan Maschinenfabrik, Berlin, Germany) operated at a rotor speed ratio of 1:1.4. Table 2 shows the formulations used for the preparation of NR composites vulcanized with sulfur. For the SWCNT-filled NR composites, reagents were mixed in two steps. First, the previously prepared SWNCTs masterbatch was initially mixed at 120 °C with stearic acid and zinc oxide for 10 min, after the temperature was lowered to 70 °C and then, the remaining 95 g of NR and the specified amounts of sulfur, MBTS, and PBN were added and mixed for 15 min. In the case of CB-filled NR composites, NR, CB, and additives were milled all together at 70 °C for 15 min. The resulting NR composites were identified as NR-S-SWCNTs or NR-S-CB. For comparison, an NR composite without using fillers was prepared as a reference and named as NR-S.

### 2.4. Preparation of NR Composites with Organic Peroxides

In the formulations with organic peroxides (T29, DCP, and T145), first, NR was added to the open two-roller mill operated at a rotor speed ratio of 1:1.4. The milling process was carried out isothermally at 70 °C for 10 min. Afterward, SWCNTs masterbatch or CB particles were added to the mill, and seven min later, the organic peroxide was added to the mixture and maintained for an additional five min. The NR composites vulcanized with each organic peroxide and containing fillers were termed under the following notation: NR-organic peroxide-filler; for example, NR-T29-SWCNTs. Similarly, an NR composite without adding fillers (Neat) was also prepared in each case and used as a reference. The composite formulations for the preparation of the NR composites vulcanized with organic peroxides are described in Table 3.

### 2.5. Vulcanization–Compression Molding Process

Vulcanization of sample plates was performed by compression molding; the NR composites obtained by the milling process were placed in a 200 mm × 200 mm × 2 mm steel mold at high temperature and compressed under 3 × 10^4^ kg cm^−2^ of pressure using a thermostated hydraulic press, Model 0230H-X4A (PHI, City of Industry, CA, USA). Temperatures and vulcanization times were set according to the vulcanization agent as described below. Once the desired vulcanization times (*t.v*.) were reached, the sample plates were placed in a hydraulic press and compressed at 3 × 10^4^ kg cm^−2^ at room temperature controlled by running water through the platens, in order to allow cooling of the NR composite plates. 

### 2.6. Determination of Temperature and Time (t.v.) of Vulcanization of NR Composites

The effect of adding organic peroxides and fillers such as CB and SWCNTs on the vulcanization of NR was evaluated. As previously mentioned, three organic peroxides: T29, DCP, and T145, were selected as cross-linking promoters; for comparison, sulfur was also considered as a cross-linking agent. Thermal decomposition of organic peroxides has been observed when they are exposed to heat energy through the vulcanization process; organic peroxides generate free radicals resulting in the cross-linking of unsaturated bonds during the vulcanization of NR. The thermal decomposition process of most organic peroxides follows first-order kinetics with a degradation rate constant *k_d_* [30]. The activation energy associated with the thermal decomposition together with the free radical half-life (*t*_1/2_) were calculated according to the following equations:

(1) $$k_{d}=Ae^{-\frac{E_{a}}{RT}}$$

(2) $$t_{1/2}=\frac{\ln2}{k_{d}}$$

where *k_d_* is the decomposition rate constant, *A* is the Arrhenius pre-exponential factor, *E_a_* corresponds to the activation energy of thermal decomposition of the organic peroxide, *R* is the universal gas constant, and *T* is the temperature at which the thermal decomposition occurs, i.e., the vulcanization temperature.

The vulcanization temperatures were selected according to the processing conditions suggested by the organic peroxide supplier, being 129, 160, and 173 °C for T29, DCP, and T145, respectively. The theoretical vulcanization times for each organic peroxide were calculated according to the reported decomposition rate constant [30] at the selected temperature and using Equation (2). We assumed that the vulcanization times (*t.v.*) were close to the peroxide half-life (*t_1/2_*) since the lifetimes of the free radicals were in the order of milliseconds. To compare the theoretical vulcanization times, the experimental vulcanization times (*t.v*.) of the NR composites were evaluated by oscillatory rheometric measurements using an oscillatory rheometer, Model MCR 301 (Anton Parr, Ashland, VA, USA).

### 2.7. Characterization Techniques

#### 2.7.1. Electron Microscopy

Analysis by high-resolution transmission electron microscopy (HR-TEM) of SWCNTs was performed in a FEI-TITAN 80–300 kV microscope operating at 120 kV. Microstructural analysis of composites was conducted on the cryofractured surfaces of specimens using a field-emission scanning electron microscope (FE-SEM) JSM 7401F (JEOL, Peabody, MA, USA) under an acceleration voltage of 5 kV.

#### 2.7.2. Mechanical Properties

For tensile strength determinations of the NR composites, dumbbell-shaped samples were prepared from the sample sheets following the ASTM D-412 standard method. For tearing measurements, the type C samples were prepared according to the ASTM D-624 standard method. All test specimens were placed into a conditioning chamber at 25 °C ± 1 °C and 50% relative humidity for 24 h before testing. Tensile and tearing tests were done in a universal testing machine, Model SFM-120 (United Testing Systems Inc., Fullerton, CA, USA) according to the specified ASTM standard method in each case. Hardness measurements of the NR composites were determined with a Shore A durometer according to the ASTM D-2240 standard method. The reported values of mechanical properties for each sample were averages obtained from the measurements performed in different specimens.

#### 2.7.3. Raman Spectroscopy

With the purpose to examine the interactions between the free radicals generated from the NR vulcanization with organic peroxides and SWCNTs, the NR composites were evaluated by Raman spectroscopy. Measurements were performed in a Raman microscope Model Xplora (Horiba Corp., Kyoto, Japan) equipped with a laser light source of 785 nm and 10× objective. Raman spectra were obtained in the range 100–4000 cm^−1^ with an acquisition time of 30 s.

### 2.8. Determination of Average Molecular Weight between Cross-Link Points ($\bar{M_{c}}$)

To evaluate the degree of vulcanization of the NR composites, calculations of average molecular weight between cross-link points ($\bar{M_{c}}$) by using the solvent swelling method were performed. One gram of the vulcanized rubber was immersed in benzene for 24 h; subsequently, the sample was extracted, dried, and weighted. The ratio between the volumetric swelling and the degree of vulcanization is given by the Flory–Rehner equation [31]:

(3) $$-\left[\ln\left(1-V_{r}\right)+V_{r}+\chi V_{r}^{2}\right]=\frac{\rho V_{s}V_{r}^{1/3}}{\bar{M_{c}}}$$

where *V_r_* corresponds to the rubber volume fraction in the swollen rubber, *χ* is the Flory–Huggins interaction parameter between rubber and solvent, *ρ* is the rubber density without additives (g cm^−3^), *V_s_* is the solvent molar volume (cm^3^ (g mol)^−1^), and $\bar{M_{c}}$ refers to the average molecular weight between cross-link points (g (g mol)^−1^).

## 3. Results and Discussion

### 3.1. Analysis of NR Vulcanization Process

**Figure 2:**
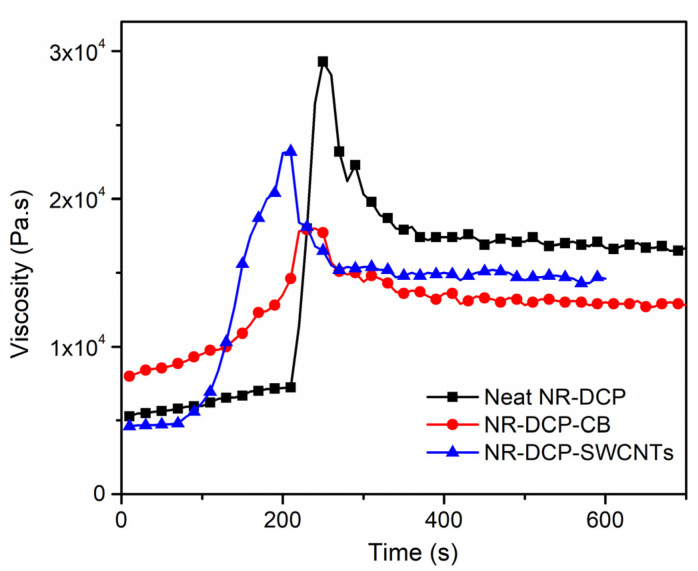
Curing curves of the NR composites using DCP at 160 °C.

To analyze the influence of the organic peroxides and fillers such SWCNTs and CB on the vulcanization of the NR composites, curing curves for each NR formulation were obtained. In order to illustrate this effect, the viscosity vs. time curves obtained for NR-SWCNTs composites using DCP at 160 °C are depicted in Figure 2. As observed, three zones can be distinguished in a typical vulcanization curve. The first one, at low viscosity values, is defined as an induction zone where the free radicals are forming, and the earlier reactions take place. The second zone corresponds to the curing stage, which is characterized by an increase in the viscosity of the elastomer; during this time, most of the cross-linking reactions occur. The vulcanization time (*t.v*.) is the time at which the highest viscosity value is reached on the vulcanization curve. The third zone is defined as an over-curing or maturation stage due to an excess of free radicals; in this region, a decrease in viscosity can be observed since the applied torque, which can lead to a certain degree of degradation of the NR. As shown, the curing curve of neat NR-DCP composite exhibits a relatively extended induction zone maintaining viscosity values almost constant by around 200 s; after, it suddenly reaches its highest viscosity value at 252 s. On the other hand, when SWCNTs are added to the formulation, a significant decrease in induction time is observed, producing that the curing peak of NR occurs at lower vulcanization time and at lower viscosity values compared to the NR composites without fillers. As for the NR-DCP-CB composite, the vulcanization curve shows a much more gradual tendency toward the maximum point of curing, which is recorded at a higher *t.v*. value than the NR-DCP-SWCNTs composite and at a lower viscosity value compared to the neat NR-DCP and NR-DCP-SWCNT composites.

The experimental vulcanization times (*t.v*.) were obtained from the analysis of the curing curves of each NR formulation; results are listed in Table 4. As shown in this table, good agreement is obtained between the calculated and the experimental *t.v.* values of the neat NR formulations. When NR was filled with SWCNTs or CB, a reduction in the *t.v*. value is observed in all cases. In this set of composites, a remarkable 50% reduction in *t.v*. (from 600 to 300 s) was found when sulfur was used as the vulcanization promoter. Similar results are found for the NR filled with SWCNT composites using organic peroxides as cross-linking agents, recording a notable reduction in *t.v*. of up to 31.7% (for T29) and 22.4% (for DCP) compared to the neat NR composites; such a reduction in *t.v*. occurs in the following order T29 > DCP > T145. We suggest that whenever SWCNTs are used as filler for NR, it is necessary to consider the decrement in *t.v.* to avoid an over-cure when the NR is processed. The reduction in *t.v.* could be considered as an acceleration of the vulcanization reaction and be attributed to two possible effects: (i) the generation of free radicals due to the thermal decomposition of the organic peroxide or accelerator (when sulfur is used—i.e., MTBS); here, the heat transfer can drive the extent of the radical generation. When the heat is better transferred to the elastomer, an acceleration in the vulcanization reactions will take place leading to the reduction in *t.v*. Based on this, we suppose that such behavior is promoted by the high thermal conductivity of SWCNTs (2000 W (mK)^−1^) [32], which can generate hot points between the nanotubes in the NR surroundings, and ii) the second effect can be attributed to the chemical reaction involved during the vulcanization process; it is well known that the SWCNTs can react with the free radicals [33]; thus, the heat generated by this reaction can influence the reduction in *t.v*. For its part, the moderate acceleration for the vulcanization reaction of the NR-CB composites which experience the same reduction in *t.v* and tendency: T29 > DCP > T145, is probably due to the lower thermal conductivity of CB (0.215 W (mK)^−1^) compared to the thermal conductivity of SWCNTs [34].

### 3.2. Microstructural Analysis

**Figure 3:**
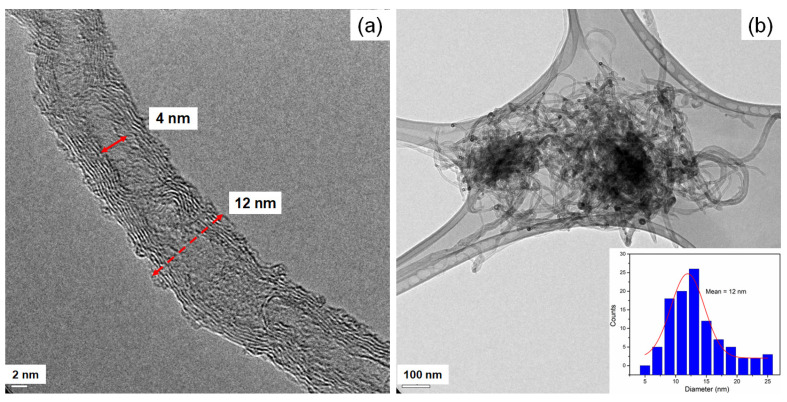
HR-TEM images of SWCNTs. (**a**) Bright-field TEM image showing the inner and outer diameter size of SWCNTs, (**b**) HR-TEM of dispersed SWCNTs. The inset in (**b**) shows the diameter size distribution of SWCNTs.

**Figure 4:**
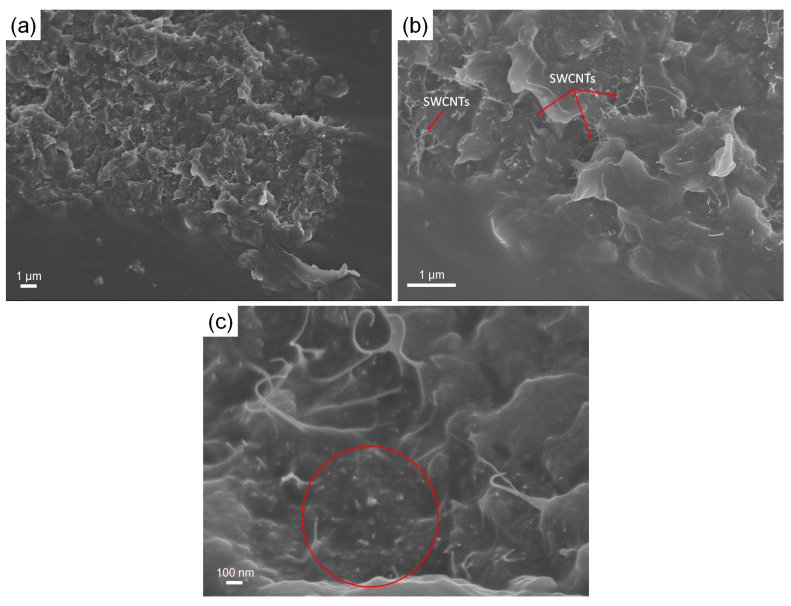
SEM images of NR-DCP-SWCNTs composite at different magnifications. (**a**) Distribution of SWCNTs into NR matrix, (**b**) red arrows show individualized and dispersed SWCNTs, (**c**) the red circle shows well-embedded SWCNTs in the matrix.

To analyze the microstructure of SWCNTs used in this work, HR-TEM images were obtained, as depicted in Figure 3. As observed, the original pristine SWCNTs show bundles with diameters in the micron range due to strong interactions among SWCNTs by the Van der Waals forces, and diameter size distribution close to 10 nm. On the other hand, the micrographs of the cryofractured surface of the NR-SWCNTs composite cross-linked with DCP, obtained at different magnifications (Figure 4), exhibit good dispersion state of SWCNTs, which are well embedded within the NR matrix (Figure 4a). Additionally, it is possible to observe individualized SWCNTs (Figure 4b) that show not only a physical interaction, but also interaction with the rubber phase (Figure 4c). Regarding the dispersion method used in our composites, we consider that it achieved an apparently good state of dispersion of carbon nanostructures in the NR and bundles are not easily appreciated.

### 3.3. Mechanical Properties of NR Composites Using Different Cross-Linking Agents

#### 3.3.1. Tensile Properties

**Figure 5:**
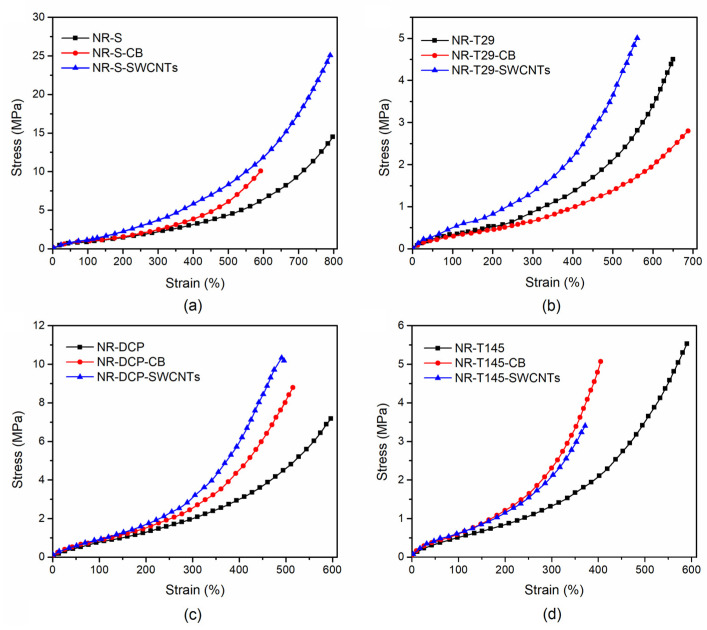
Stress–strain curves of the NR composites vulcanized with different cross-linking agents: (**a**) sulfur, (**b**) T29, (**c**) DCP, (**d**) T145 and incorporating CB or SWCNTs as fillers.

The mechanical performance of NR is highly dependent on (i) the shape and dispersion of the filler throughout the material, (ii) the dispersion of the cross-linking agent in the polymer chains that promotes the cross-linking, and (iii) the nature and degree of the resulting cross-linking network. Composite plates were prepared and vulcanized by compression molding using the vulcanization temperatures and *t.v*., listed in Table 4, with the purpose to evaluate the effect of CB, SWCNTs, and the cross-linking reaction on the mechanical properties of the NR composites with different vulcanizing agents. Figure 5 illustrates the tensile stress–strain curves obtained from the NR composites vulcanized with sulfur, organic peroxides, and CB or SWCNTs as fillers. Table 5 summarizes the values of tensile strength, strain at break, and modulus (at 100 and 300% strain) measured for each NR formulation. As observed in Figure 5a, tensile stress–strain curves for the NR composites vulcanized with sulfur exhibit the highest values of tensile strength and strain at breakage (up to 800%) in comparison with the tensile curves of the NR composites vulcanized with organic peroxides (Figure 5b–d). When SWCNTs are added to NR formulations, significant increases in tensile strength, 71, 75, and 40%, are observed when comparing with the neat NR composites vulcanized with sulfur, T29, and DCP, respectively. However, in all the cases the reinforcing effect produced by SWCNTs leads to a decrease in the strain at breakage up to 37% (for example, in the NR-DCP-SWCNTs composite) besides changes in the behavior of the curves showing steeper slopes, which are clearly attributed to the stiffening of the NR matrix. Despite this, the increase in tensile strength suggests an adequate dispersion and distribution of SWCNTs and good interfacial adhesion between SWCNTs and the elastomer matrix, as shown in the SEM images [20].

As for the NR-CB composites, the presence of CB particles does not produce increases in tensile strength of the NR composites vulcanized with different agents; nevertheless, decreases in the strain at breakage and steeper slopes in the curves compared to the NR composites without fillers can be observed, although, to a lesser extent than with SWCNTs, it is also related to the increase in the mechanical stiffness of the NR composites, as described below.

**Figure 6:**
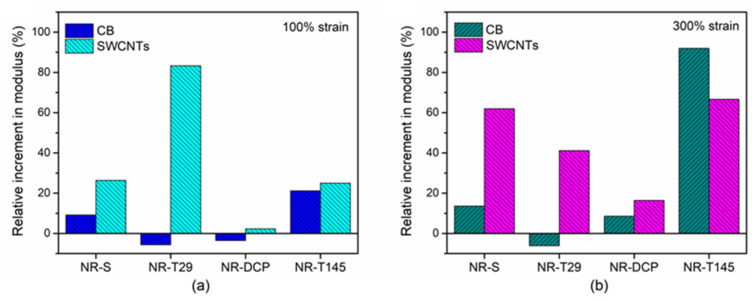
Relative increments in modulus at (**a**) 100 and (**b**) 300% strain for the NR composites vulcanized with different cross-linking agents and incorporating CB or SWCNTs as fillers.

Figure 6 shows the comparison between the relative increments in modulus at 100 and 300% strain for all the NR composites samples with different vulcanization agents and incorporating CB or SWCNTs. Relative increments in modulus for each set of samples were calculated based on the modulus value obtained in the neat NR composite (NR-S, NR-T29, NR-DCP, or NR-T145). Remarkable increases in modulus for the NR-SWCNTs composites are observed when compared with the neat NR or NR-CB composites. For the NR-S composites with SWCNTs, increments of 26% (at 100% strain) and 62% (at 300% strain) compared to the neat NR-S sample are found. On the other hand, moduli of the NR composites vulcanized with organic peroxides also show significant increments after SWCNTs incorporation; for example, the NR-T29 composites increase 83% and 41% at 100% and 300% strain, respectively, and the NR-T145 composites recorded 25% and 67% enhancement at the same conditions. However, when DCP was used as a cross-linker, a moderate increment in modulus value for SWCNTs is observed, showing a maximum increment of 16% when compared with the neat formulation; nonetheless, it should be noted that the values measured for modulus at 100 and 300% strain for the NR-DCP composites (see Table 5) are very close to those obtained for the NR-S composites. 

For NR composites containing CB as filler, increments in modulus for most of the samples are less significant than those observed in the NR composites with SWCNTs. Particularly, in the NR composites vulcanized with organic peroxides (T29 and DCP), the CB addition causes a detrimental effect in modulus at 100% strain, unlike the tendency observed in the NR-T145 composites, where the CB particles produce a notable increase in stiffness for the NR formulations.

#### 3.3.2. Tear Strength and Hardness

The tear strength and hardness were additionally evaluated for these materials. The values obtained for the NR composites vulcanized with different cross-linking agents and in the presence of CB or SWCNTs are shown in Table 6. In general, the vulcanization using sulfur produces NR composites with higher tear strength in comparison with the use of organic peroxides. From SWCNTs addition to the neat NR formulations, increments in the tear strength from 4 to up to 27% for most of the composites are observed; however, the effect of reinforcement brought from the SWCNTs incorporation is less significant than that observed in tensile strength and modulus. As for the NR-CB composites, except for the NR-T29-CB composite, the tear strength diminishes when compared with the neat NR composites. 

Regarding the hardness measurements, it can be observed that the NR-DCP composites exhibit the highest values of hardness among all the samples analyzed in the present work. Similar to the tear strength values, the SWCNTs addition to either the sulfur or organic peroxides formulations modestly increases the hardness in a range of 3 to 10% compared to the neat NR composites. In the case of the NR-CB composites, the increases in hardness are lower than those found for the NR-SWCNTs composites. Considering that the differences between the hardness values obtained for the neat NR and NR with fillers for a given cross-linked agent are relatively small, it would be difficult to establish any behavioral tendency for the produced NR composites.

### 3.4. Evaluation of the Degree of Vulcanization of NR Composites

One typical method to evaluate the degree of vulcanization in elastomers is the determination of the average molecular weight of the rubber segments between neighboring cross-links, $\bar{M_{c}}$, [31,35]; a lower value of $\bar{M_{c}}$ implies higher vulcanization and improvement in the mechanical properties. In that sense, the $\bar{M_{c}}$ values for the NR composites vulcanized with different cross-linking agents and adding CB or SWCNTs were calculated and included in Table 6. As observed, the $\bar{M_{c}}$ values obtained for the NR composites vulcanized with organic peroxides are higher than those of the NR-S composites; such behavior can be attributed to dissimilarities in the type and number of bonds formed during the cross-linking process. Specifically, the addition of SWCNTs to NR formulated with T29 and DCP leads to an important reduction in the $\bar{M_{c}}$ values in comparison with those of the neat NR formulations and the NR-CB composites; this is indicative of a major degree of cross-linking derived from the existence of multiple interactions between SWCNTs and free radicals generated during the vulcanization process. Based on the above, it is possible to confirm that the use of SWCNT at contents <1 phr can increase the degree of vulcanization of NR mainly when organic peroxides are used as cross-linking agents, thus leading to the enhancement of mechanical properties of the developed NR composites, as previously discussed.

**Scheme 1:**
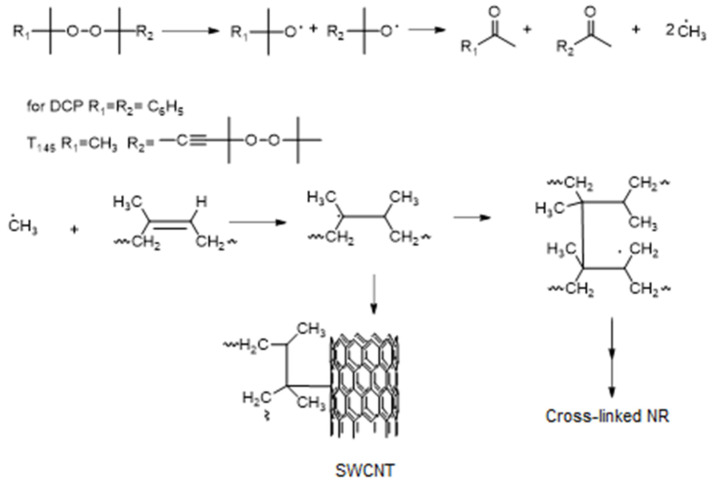
Possible vulcanization reaction mechanism of NR based on the studied organic peroxides.

It is interesting to consider that the type of bonds formed during the cross-linking process depends on the curing agents. In the case of sulfur, mono- (C-S-C), di- (C-S_2_-C), and polysulfidic (C-S_x_-C) bonds can be formed [4,36,37]. It is well known that the longer the bond that links the rubber molecules, then higher will be the mobility between polymeric chains, and thus, the final rubber compound will be more flexible. In contrast, organic peroxides generate exclusively C-C bonds less flexible than C-S_x_-C chemical bonds. As a result, one expects that the tensile failure strain will be higher in materials with C-S_x_-C bonds and lower in materials vulcanized with exclusive C-C bonds. This can explain the lower values of tensile strength obtained in the NR composites vulcanized with organic peroxides. Furthermore, it is well accepted that the interactions between nanotubes and rubber molecules occur through entanglements and classical nonbonded interactions [38]; however, the reduction observed in the vulcanization time of the NR-SWCNTs composites in conjunction with the increments in moduli, tensile strength, and reduction in the strain at breakage (Table 5) lead us to suppose that an interaction between the NR and the SWCNTs certainly occurred. A key condition for such an interaction to occur is an efficient dispersion of SWCNTs in the rubber matrix, which was accomplished by the previous SWCNTs dispersion in the masterbatch and the subsequent milling process with the components of the formulation. In that sense, we suggest a grafting reaction between the rubber macroradicals generated during the vulcanization process with the surface of SWCNTs. The free radicals generated at the NR segments may interact with the walls of the nanotube resulting in a reduction in the mobility of the NR chains, thus producing a more rigid polymer network. Scheme 1 shows a possible vulcanization reaction mechanism of NR using the studied organic peroxides, including the possible reaction between the free radicals generated due to the thermal decomposition of the organic peroxide with the SWCNT surface.

Additionally, the differences found in mechanical properties for the peroxides used are related to the cross-linking densities obtained in each case. This can be explained by the different reaction mechanisms that occurred, which are mainly based on the type and the free radical half-life (*t*_1/2_). In general, organic peroxides first undergo homolytic cleavage to form primary radicals, which can be fragmented into secondary radicals, as shown in Scheme 1, modifying the chemistry of the generated radicals. Such is the case of DCP, where its homolytic cleavage leads to the formation of primary cumyloxy radicals, which can experience a beta scission to form secondary methyl radicals and acetophenone [39,40,41]. Methyl radicals are smaller and less sterically hindered, so that they are more accessible to reactive functional centers on rubber chains; in addition, they have a lower half-life, which changes the physical properties of vulcanizates and promotes a major degree of cross-linking, lower values of $\bar{M_{c}}$ and, in consequence, better mechanical properties, as shown Table 5 and Table 6. Apparently, this mechanism is favored when SWCNTs are employed in the formulations, resulting in lower values of $\bar{M_{c}}$ due to the multiple interactions among the sp^2^ carbon bonds of SWCNTs and free radicals generated during the vulcanization process (Scheme 1), which produce a more closed network with a larger number of cross-link points.

Considering the other two peroxides (T29 and T145), a similar mechanism can occur; however, the mechanical properties observed in these composites were lower than those for DCP composites. These results can be attributed to the occurrence of reactions when adding peroxide radicals to the double bonds of unsaturated rubber chains or abstraction of reactive allylic hydrogen atoms [41]. Both mechanisms can proceed concurrently and be highly competitive, leading to the formation of multiple chain bonds, which can generate clusters that decrease the resulting mechanical properties.

### 3.5. Raman Spectroscopy of NR-SWCNTs Composites

**Figure 7:**
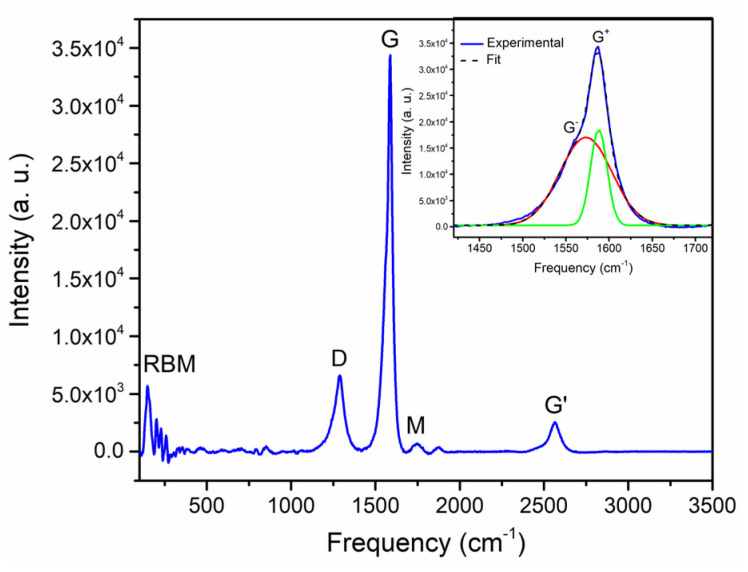
Raman spectrum of pristine SWCNTs (semiconducting). The inset shows the deconvolution of the G-band into gaussian components performed to show the weak double resonance features on G-band associated with the M-band. Here, the blue solid line corresponds to experimental data, the dashed line to the curve fitting, and the red and green solid lines indicate the computed fitting curves.

Raman spectroscopy has been used as an efficient method to study the effect of SWCNTs on NR in composites [17,18]. Figure 7 shows the Raman spectrum of the pristine SWCNTs employed as filler in the NR composites. As observed, the characteristic absorption bands of SWCNTs comprise the radial breathing mode (RBM) at low frequencies in the range 100–250 cm^−1^, the tangential G-band at 1585 cm^−1^, and several weak features such as the D-band at 1310 cm^−1^ related to defects in the nanotube structure [42,43] and the M-band near 1750 cm^−1^ (an overtone of the out-of-plane infrared-active mode in graphite) [44]. The presence of such absorption bands matches the semiconductor nature of SWCNTs. Additional evidence of the semiconductor nature of the SWCNTs employed in the present work is the distortion of the line shape of the G-band (inset in Figure 7) attributed to the $G^{+}$ and $G^{-}$ features. The first feature ($G^{+}$) is associated with carbon atom vibrations along the nanotube axis (longitudinal optic phonon mode, which appears at 1585 cm^−1^) and the second $G^{-}$ feature (at 1556 cm^−1^), which is sensitive to charge transfer from dopant additions to SWCNTs [43,45]. The G’-band is considered a second-order feature and gives information about the electronic structure of the semiconductor or metallic nature of SWCNTs.

**Figure 8:**
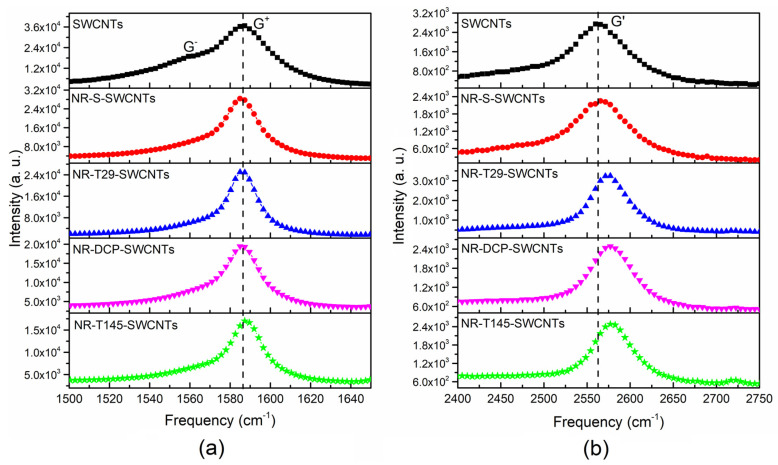
Raman spectra of (**a**) G-, and (**b**) G’-band of the NR-SWCNTs composites using different cross-linking agents.

Figure 8 displays the G- and G’-band Raman spectra of the NR-SWCNTs composites formulated with different cross-linking agents compared with the Raman spectrum of the pristine SWCNTs. As shown in Figure 8a, the intensity of the G-band for pristine SWCNTs monotonically decreases when reacting with the different cross-linking promoters; such tendency seems to be more evident in the NR-SWCNTs composites vulcanized with organic peroxides. Another important observation is that the $G^{-}$ feature of SWCNTs completely disappears once the vulcanization process with each cross-linker occurs, which can reflect the hardening of the C–C bonds as the electronic density decreases [46], probably due to the reaction between the free radicals formed during the vulcanization process with organic peroxides and the wall surface of the SWCNTs. Additional data to support such assertions can be obtained by analyzing the D-band (Figure 7); the absorption at 1310 cm^−1^ corresponds to disordered graphite structures. The D-band is activated through the first-order excitation process of sp^2^ carbons by the presence of in-plane-substituted heteroatoms, vacancies, and grain boundaries, and provides unique information on the chirality and diameter dependence [43]. This second-order feature can be used for materials characterization to probe and monitor structural modifications of the nanotube sidewalls that come from the introduction of defects and the attachment of different chemical species. It has been accepted that the sample purity or the modification degree of the nanotube sidewall can be obtained by calculating the D/G band intensity ratio (*I_D_*/*I_G_*) from the Raman spectra of SWCNT composites [47]; thus, the intensity ratios of the NR-SWCNTs composites with different vulcanization agents were calculated and listed in Table 7. For the vulcanized composites, increments in the *I_D_*/*I_G_* ratio due to increases in the D-band intensity are obtained. This result indicates a sidewall modification in the SWCNTs due to the reaction with the free radicals generated during the vulcanization process; such an interaction introduces defects and probably attaches reactive chemical species to the nanotube walls.

Additionally, Figure 8b shows a comparison of the G’-band Raman spectra of the pristine SWCNTs and the NR-SWCNTs composites. In most of the NR-SWCNTs composites vulcanized using either sulfur or organic peroxides, the G’-band exhibits decrements in intensity and notable shifts toward higher-frequency compared to the pristine SWCNTs. Shifts in the G’ absorption band for the vulcanized materials were calculated from the difference in frequency recorded for the NR-SWCNTs composites and the pristine SWCNTs (2561 cm^−1^) and included in Table 7. As can be observed, the NR-SWCNTs composites vulcanized with organic peroxides show positive shifts from 13 up to 17 cm^−1^ concerning the original G’-band frequency, which indicates an interaction between the free radicals generated during the NR vulcanization and the sidewalls of the SWCNTs. According to Zhao et al. [18], the extent of the G’-band shift can be correlated to the degree of vulcanization in elastomer composites. Based on this and from the mechanical properties and cross-linking degree obtained in our NR composites, we can confirm that the addition of a small content of SWCNTs (0.7 phr) to NR formulations favorably promotes the vulcanization of NR, mainly when organic peroxides are used as cross-linking agents; this is due to the multiple interactions between SWCNTs and the free radicals generated during the vulcanization process, which leads to considerable enhancement in physical properties of the developed NR-SWCNTs composites.

## 4. Conclusions

The effect of SWCNTs on the vulcanization process of the NR using organic peroxides was analyzed. NR composites filled with SWCNTs show a notable reduction in the *t.v.* values when compared with the neat NR composites and the NR-CB composites; this is associated with the presence of free radicals generated by thermal decomposition. We suggest that the concentration of the free radicals was increased by the high thermal conductivity of the SWCNTs. The mechanical properties of the NR composites vulcanized with different cross-linking agents exhibit increases in tensile strength, moduli, tear strength, and hardness of up to 75, 83, 27, and 10%, respectively, when the SWCNTs are used as filler in the NR composites. The lower values of strain at breakage for the NR-SWCNTs composites obtained through the vulcanization with organic peroxides suggest that the C-C bonds formed during the process promote the formation of more rigid polymer networks. The evaluation of the degree of vulcanization by the calculations of $\bar{M_{c}}$ values confirms the improvement in the vulcanization for the NR-SWCNTs composites, mainly when organic peroxides are used as cross-linking agents. The analysis by Raman spectroscopy indicates the interactions between nanotube walls, free radicals, and reactive chemical species during the vulcanization process, which introduce defects that increase the disorder (D-band) and reduce the semiconductor character (G-band) of the SWCNTs. From the experimental findings obtained for the NR composites developed in the present work, it is possible to confirm that the incorporation of SWCNTs at a relatively low content (0.7 phr) in conjunction with the use of organic peroxides for the NR vulcanization represents a potential alternative to the traditional methods used for the improvement of the physicochemical properties of NR composites.

## Figures and Tables

**Table 1:** Physical properties of organic peroxides.

Property	T29	DCP	T145
Melting point (°C)	−20	39–41	88
Boiling point (°C at 1 atm)	403.47	130	348.77
Density (g·cm^−3^)	0.895	1.56	1.26

**Table 2:** Formulations used for sulfur vulcanized NR composites.

Component	NR (phr)	CB (phr)	SWCNTs (phr)
NR	100	100	100
Zinc oxide	5	5	5
Stearic Acid	1	1	1
Sulfur	2.5	2.5	2.5
MBTS	1	1	1
PBN	1	1	1
CB	-	0.7	-
SWCNTs	-	-	0.7

**Table 3:** Formulations used for organic peroxide vulcanized NR composites.

Component	NR (phr)	CB (phr)	SWCNTs (phr)
NR	100	100	100
Organic peroxide ^1^	2.5	2.5	2.5
CB	-	0.7	-
SWCNTs	-	-	0.7

^1^ Organic peroxides used were T29, DCP, or T145.

**Table 4:** Vulcanization times (*t.v.*) obtained for the vulcanization of NR composites.

Sample	*t.v.* (s) (% Time Reduction)			
	Sulfur (160 °C)	T29 (129 °C)	DCP (160 °C)	T145 (173 °C)
Calculated ^1^	-	382	255	368
Neat	600	380	252	368
CB	480 (20)	312 (18.3)	240 (5.9)	360 (2.2)
SWCNTs	300 (50)	261 (31.7)	198 (22.4)	306 (16.8)

^1^ The half-life time of the organic peroxides (*t*_1/2_) calculated with the *k_d_* value at the processing temperatures.

**Table 5:** Values of tensile properties obtained for NR composites.

Sample ID	Tensile Strength (MPa)	Strain at Breakage (%)	Modulus (MPa)	
			100%	300%
NR-S	14.77	800	0.87	2.50
NR-S-CB	10.39	600	0.95	2.84
NR-S-SWCNTs	25.23	790	1.10	4.05
NR-T29	4.52	651	0.36	1.14
NR-T29-CB	2.82	687	0.34	1.07
NR-T29-SWCNTs	7.90	564	0.66	1.61
NR-DCP	7.25	600	0.86	2.56
NR-DCP-CB	8.77	516	0.83	2.78
NR-DCP-SWCNTs	10.14	495	0.88	2.98
NR-T145	5.52	592	0.52	1.50
NR-T145-CB	5.06	406	0.63	2.88
NR-T145-SWCNTs	3.44	372	0.65	2.50

**Table 6:** Values of tear strength, hardness, and $\bar{M_{c}}$ obtained for NR composites.

Sample ID	Tear Strength (N m^−1^)	Hardness	$\bar{M_{c}}$ g (g mol)^−1^
NR-S	68.6	30.4	7353
NR-S-CB	61.2	31.6	7353
NR-S-SWCNTs	71.4	33.6	9434
NR-T29	6.3	17.2	64,831
NR-T29-CB	7.7	16.5	101,555
NR-T29-SWCNTs	8.0	18.2	17,752
NR-DCP	51.5	35.4	8571
NR-DCP-CB	38.2	37.6	108,042
NR-DCP-SWCNTs	55.1	37.4	7353
NR-T145	36.2	23.3	19,584
NR-T145-CB	15.3	21.2	48,723
NR-T145-SWCNTs	23.4	24	29,215

**Table 7:** *I_D_*/*I_G_* ratio of the NR-SWCNTs composites with different vulcanization agents.

NR-SWCNTs Composites	*ID*/*IG* Ratio	Frequency, *δ* $\left[\delta_{{G^{\prime }}_{NR-SWCNTs}}-\delta_{{G^{\prime }}_{SWCNTs}}\right]$ (cm^−1^)
SWCNTs (pristine)	0.2452	0
NR-S-SWCNTs	0.3778	5
NR-T29-SWCNTs	0.3648	13
NR-DCP-SWCNTs	0.3195	16
NR-T145-SWCNTs	0.3520	17

## Data Availability

Not applicable.
